# Mental Distress Among Youths in Low-Income Urban Areas in South America

**DOI:** 10.1001/jamanetworkopen.2025.0122

**Published:** 2025-03-05

**Authors:** Carlos Gómez-Restrepo, Francisco Diez-Canseco, Luis Ignacio Brusco, Maria Paula Jassir Acosta, Natividad Olivar, Fernando Luis Carbonetti, Liliana Hidalgo-Padilla, Mauricio Toyama, José Miguel Uribe-Restrepo, Nelcy Rodríguez Malagon, David Niño-Torres, Natalia Godoy Casasbuenas, Diliniya Stanislaus Sureshkumar, Catherine Fung, Victoria Bird, Craig Morgan, Ricardo Araya, James Kirkbride, Stefan Priebe

**Affiliations:** 1 Department of Psychiatry and Mental Health, Pontificia Universidad Javeriana, Bogotá, Colombia; 2 Department of Clinical Epidemiology and Biostatistics, Pontificia Universidad Javeriana, Bogotá, Colombia; 3 Hospital Universitario San Ignacio, Bogotá, Colombia; 4 CRONICAS Center of Excellence in Chronic Diseases, Universidad Peruana Cayetano Heredia, Lima, Perú; 5 Department of Psychiatry and Mental Health, School of Medicine, Universidad de Buenos Aires, Buenos Aires, Argentina; 6 Unit for Social and Community Psychiatry, WHO Collaborating Centre for Mental Health Service Development, Queen Mary University of London, London, United Kingdom; 7 Health Service and Population Research, King’s College London, London, United Kingdom; 8 Centre for Global Mental Health, Institute of Psychiatry, Psychology, and Neuroscience, King’s College London, London, United Kingdom; 9 Division of Psychiatry, University College London, London, United Kingdom; 10 Centre for Psychosocial Medicine, University of Hamburg, Hamburg, Germany

## Abstract

**Importance:**

Improving mental health of young people is a major societal challenge, particularly among the high numbers of young people living in deprived urban areas.

**Objective:**

To identify factors associated with depression and anxiety among young people in deprived urban areas in South America.

**Design, Setting, and Participants:**

This case-control study recruited adolescents (age 15-16 years) and young adults (age 20-24 years) from education and community settings in deprived areas in Bogotá, Colombia; Buenos Aires, Argentina; and Lima, Peru, between April 2021 and November 2022. Participants who met threshold criteria for self-reported depression or anxiety. Community controls who did not meet the criteria were identified.

**Exposures:**

Sociodemographic characteristics, stressful life events before and during the past year, substance use, social capital, sports and arts activities, social media engagement.

**Main Outcomes and Measures:**

Depression, assessed using the 8-item Patient Health Questionnaire (range, 0-24, with higher scores indicating greater symptom severity), and anxiety, assessed using the 7-item Generalized Anxiety Disorder questionnaire (range, 0-21, with higher scores indicating greater symptom severity), were defined by threshold scores higher than 9. Various factors were compared between groups with and without anxiety and depression in multivariable logistic regression, testing for interactions by age group.

**Results:**

Of 2402 analyzed participants, 1560 (64.9%) were female, 1080 (45.0%) were adolescents, and 1322 (55.0%) were young adults; 1437 (59.8%) met the criteria for depression and/or anxiety, and 965 (40.2%) were controls. In a multivariable model, female gender (OR, 1.99 [95% CI, 1.65-2.40), more than 2 stressful life events in the previous year (OR, 1.67 [95% CI, 1.40-2.01]), more than 7 stressful life events before the previous year (OR, 1.52 [95% CI, 1.27-1.81), lifetime consumption of sedatives (OR, 2.26 [95% CI, 1.65-3.14]), participating in arts activities in the past 30 days (OR, 1.22 [95% CI, 1.01-1.48]), and stronger engagement with social media (OR, 1.59 [95% CI, 1.34-1.89]) were independently associated with increased odds of depression and anxiety, while sports activities were associated with reduced odds (OR, 0.80 [95% CI, 0.67-0.96]). The odds of having depression and/or anxiety symptoms associated with lifetime use of sedatives were higher among adolescents (OR, 6.54 [95% CI, 3.33-14.27]) than among young adults (OR, 2.54 [95% CI, 1.79-3.66]) (*P* = .01 for interaction).

**Conclusions and Relevance:**

In this case-control study, female gender, stressful life events, substance use, arts activities, and social media engagement were associated with greater odds of depression and anxiety, while sport activities were associated with lesser odds. The findings suggest that policies for improving mental health in deprived urban neighborhoods in South America and related research should consider similar factors associated with mental distress in adolescents and young adults.

## Key Points

**Question:**

What factors are associated with symptoms of depression and anxiety among adolescents and young adults in deprived urban areas in South America?

**Findings:**

In this case-control study of 2402 adolescents and young adults, female gender, more stressful life events, lifetime sedative consumption, participation in arts activities, stronger social media engagement, and absence of sports activities were independently associated with depression and anxiety symptoms.

**Meaning:**

Policies for improving youth mental health in deprived South American urban areas may consider aiming to reduce substance use and social media engagement and to increase participation in sport activities.

## Teaser

This case-control study investigates factors associated with symptoms of depression and anxiety among adolescents and young adults residing in deprived urban areas in South America.

## Introduction

Reducing mental health problems in young people is a major societal challenge.^1,2^ About 70 million individuals aged 15 to 24 years currently live in South America, where rapid urbanization has resulted in high numbers of young people living in deprived urban areas. These individuals are frequently exposed to economic hardship, violence, and other social and economic inequities,^3^ commonly regarded as risk factors for mental health disorders, and studies suggest a relatively high prevalence of depression and anxiety among young people.^4,5^ Policies to improve mental health in young people living in such areas should consider both potential risk and protective factors.

Worldwide evidence suggests that female gender, poor health during childhood, special educational needs, parental separation, parental or family history of depression, problems with interpersonal relationships, stressful life events, and living in a household receiving welfare are risk factors for poor mental health.^6^ Specifically in South America, experiencing crime, living in an urban area, and lower educational level have been found as additional risk factors.^7,8,9,10^ Further behavioral risk factors include substance use disorder and an intensive engagement with social media as well as the lack of engagement in extracurricular activities, such as sports, arts, and community programs. These factors have been associated with risk in various studies, although not yet in South America.^11,12,13^

Against this background, we aimed to identify factors associated with depression and anxiety in young people in deprived neighborhoods in 3 large South American cities, focusing on symptom levels in a population-based sample rather than on diagnostic entities. We considered various factors, including sociodemographic characteristics, previous experiences, substance use, engagement in arts and sports activities, and social media engagement. Given that the definition of youths by the United Nations^14^ covers an age range with different stages of personal and professional development, we also explored whether the identified factors varied between adolescents aged 15 and 16 years and young adults aged 20 to 24 years.

## Methods

### Study Design and Setting

We conducted a case-control study of 2 groups of young people (15-16 years of age and 20-24 years of age) living in the 50% of areas with lower income in Bogotá, Colombia; Buenos Aires, Argentina; and Lima, Peru. The study was part of a larger research program.^15^ The study protocol was approved by the institutional review boards of Universidad de Buenos Aires and Pontificia Universidad Javeriana, Universidad Peruana Cayetano Heredia and the research ethics committee of Queen Mary University of London. We obtained written informed consent from the participant or the parent or legal tutor in the case of adolescents (and assent from the adolescents). This study followed the Strengthening the Reporting of Observational Studies in Epidemiology (STROBE) reporting guideline for case-control studies.

Recruitment and assessment of participants was conducted between April 2021 and November 2022, during the COVID-19 pandemic. During the initial phases of the data collection (the first 10-15 months of the 19-month period of data collection), there were social restrictions in place that varied both between cities and within each city over time.

### Participants and Recruitment Strategies in 3 Cities

The inclusion criteria for recruitment were (1) living in the city’s 50% lower-income areas, (2) being 15 to 16 or 20 to 24 years of age, and (3) providing written informed consent. For identifying the 50% lower-income areas in each city, we ranked all districts according to nationally available needs indices. The method is described in more detail in the protocol article.^15^

Exclusion criteria for all participants were (1) a diagnosed serious mental disorder (psychosis, bipolar disorder, or schizophrenia), (2) a diagnosed learning disability, (3) illiteracy, and (4) lack of capacity to provide informed consent for young adults and assent for adolescents. The recruitment strategy varied across the 3 cities, reflecting different regulations and practical options (details are given in eAppendix 1 in Supplement 1).

### Sample Size

As outlined in the sample size calculation in the protocol,^15^ we aimed to recruit a total of 2040 participants across the 3 cities. This aim was to enable us to include 340 young people in each city meeting our criteria for symptoms of depression and anxiety.

### Procedures

The informed assent and consent processes were carried out either by telephone with an audio recording, by sending a photograph or scan, or via an online form. Signatures were collected digitally or during an in-person meeting. All participants were reimbursed for their participation, equivalent to $10.

Each participant was asked to complete a battery of questionnaires. The whole procedure lasted between 30 and 60 minutes.

### Case-Control Status

Symptoms of depression and anxiety were assessed on the self-rated 8-item Patient Health Questionnaire (PHQ-8)^16^ (range of scores, 0-24, with higher numbers indicating greater symptom severity) and the General Anxiety Disorder 7-item (GAD-7)^17^ (range of scores, 0-21, with higher numbers indicating greater symptom severity). Participants were then categorized into cases and controls according to their symptom scores. We used a score of 10 or more on both the PHQ-8 and the GAD-7 as the cut-off point for symptoms.^16,17^ Individuals meeting the threshold criterion on either or both scales were classified as cases and the others as controls.

### Studied Factors

Sociodemographic data included age (15-16 or 20-24 years), gender, participant-reported history of mental health treatment of mother and father, educational level (in analysis, dichotomized as incomplete secondary education and below or full secondary education and above), relationship status, having an own bedroom, and main occupation status. Gender was ascertained by self-report and included female, male, and other, which was not further subcategorized because of the small number of individuals in this category. Stressful life events were measured separately for the past year and the period before that on a scale modified from the Adolescent Appropriate Life Events Scale^18^ by the Resilience, Ethnicity, and Adolescent Mental Health (REACH) research program,^19^ with 30 items for each period (eMethods 1 in Supplement 1). The 2 variables were dichotomized using the median (1 stressful life event in the previous year and 6 stressful life events in the period before the previous year).

Substance use was assessed with questions from the Alcohol, Smoking and Substance Involvement Screening Test scale.^20^ Participants reported whether they had ever used any of 10 different types of nonprescribed psychoactive substances (each type yes or no) (eMethods 2 in Supplement 1 gives details).

Social capital was measured on the shortened version of the Adapted Social Capital Assessment Tool^21^ using only the score on structural social capital, categorized into low (0-1), medium (2-3), and high structural social support (4-6). We focused on structural social capital, as we assumed data on cognitive social capital to be more directly influenced by symptoms.

Sports and arts activities in the past 30 days were assessed with 11 questions each. The answers were collapsed into 1 dichotomous option each for sports and arts activities (yes or no).

Social media engagement in the past 30 days was assessed on a version of the Multidimensional Facebook Intensity Scale,^22^ adapted from the REACH program.^19^ It measures the level of engagement with social media on a Likert scale (1 [strongly disagree] to 5 [strongly agree]). Scores higher than the median of 3 were considered to indicate stronger engagement.

### Missing Data

The multiple imputation by chained equations with logistic regression method was used to impute missing data independently for each variable with less than 20% missing values. Variables with more than or equal to 20% missing values were excluded.

### Statistical Analysis

Descriptive statistics are presented with frequencies, percentages, medians, means, and SDs, as appropriate. As expected, there were large differences between the 2 age groups in terms of relationship status, educational level, and main occupation. Given that age was the main determinant of these differences, we did not consider these factors further in the analysis. Following multiple imputation by chained equations, we performed univariable and multivariable logistic regressions on the imputed datasets, with odds ratios (OR) and 95% CIs. The multivariable regression model included only factors that showed an association with the outcome at *P* < .10. The multicollinearity of the multivariable model was assessed using the condition index. Finally, variables that remained associated with the outcome in the multivariable model at a threshold of a 2-sided *P* < .05 were tested for their interaction with age group using contingency tables and analyzing the difference in ORs between the groups.

Statistical modeling used the R, version 4.3.0 statistical programming language and RStudio 2023 (Posit Software, PBC). Descriptive statistics were conducted in Stata 17 (StataCorp LLC).

## Results

A total of 2402 participants were recruited. Of these, 1080 (45.0%) were adolescents; 1322 (55.0%), young adults; 1560 (64.9%), female; 815 (33.9%), male; and 24 (1.0%), other gender (data were missing for 3). The mean (SD) scores for PHQ-8 and GAD-7 scales were 10.0 (5.97) and 8.52 (4.92), respectively. Overall, 1437 participants (59.8%) had scores of 10 or more on the PHQ-8, GAD-7, or both and were therefore classified as cases with symptoms of depression and/or anxiety. Of all cases, 454 (31.6%) showed only symptoms of depression, 163 (11.3%) showed only symptoms of anxiety, and 820 (57.1%) had symptoms of both. We recruited 965 control participants (40.2%). The presence of symptoms of depression and of anxiety were strongly correlated^23^ in the total sample (Cramer V, 0.51 [95% CI, 0.47-0.55]; *P* < .001), justifying our decision to treat the scores in both symptom scales within a combined variable. The recruitment and selection process and flow of participants throughout the study are shown in the Figure.

**Figure.**
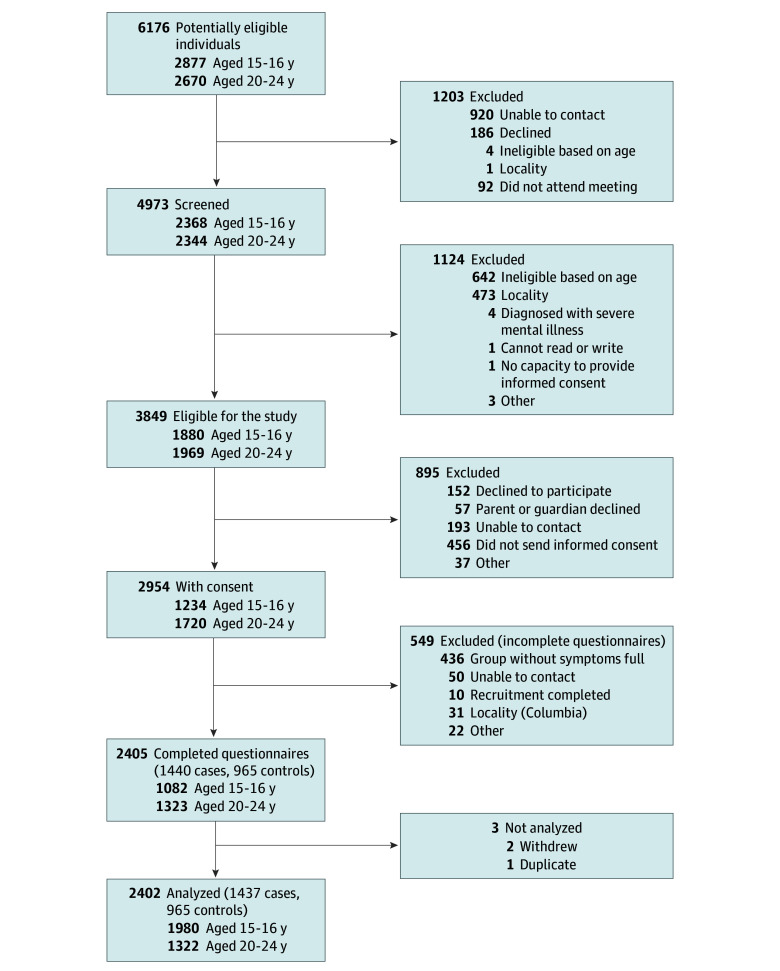
Flow Diagram of Adolescents and Young Adults Included in the Study

Regarding missingness, for the variable “mother has received mental health treatment,” there were 255 cases with missing data (17.7%) and 118 controls (12.2%); these were imputed as described in the Methods. Only 1 variable, “father has received mental health treatment,” had 20% or more missing values and was excluded.

### Sociodemographic Characteristics

The sociodemographic characteristics of both groups and the total sample are shown in Table 1. While the age ranges were determined by the inclusion criteria, there were overall more female participants. Most participants were in or had already completed at least secondary education. Also, most parents had completed secondary education.

**Table 1.** Sociodemographic Characteristics for the Total Sample and for Cases and Controls

Sociodemographic variable	Participants, No. (%)		
	Overall (n = 2402)	Without symptoms (n = 965)	With symptoms (n = 1437)
Age, y			
15-16	1080 (45.0)	435 (45.1)	645 (44.9)
20-24	1322 (55.0)	530 (54.9)	792 (55.1)
Gender			
Female	1560 (65.0)	533 (55.2)	1027 (71.5)
Male	815 (34.0)	428 (44.4)	387 (26.9)
Other^a^	24 (1.0)	4 (0.4)	20 (1.4)
Missing data	3	NA	NA
Relationship status^b^			
Single	1637 (68.3)	683 (70.9)	954 (66.5)
Not single	761 (31.7)	281 (29.1)	480 (33.5)
Missing data	4	NA	NA
Educational level			
No formal education	10 (0.4)	5 (0.5)	5 (0.3)
Primary education^c^	66 (2.7)	31 (3.2)	35 (2.4)
Secondary education incomplete or in progress	1112 (46.3)	452 (46.8)	660 (45.9)
Completed secondary education	242 (10.1)	113 (11.7)	129 (9.0)
Technical education incomplete or in progress	353 (14.7)	134 (13.9)	219 (15.2)
Technical education complete	136 (5.7)	61 (6.3)	75 (5.2)
University education^c^	481 (20.0)	167 (17.3)	314 (21.9)
Other	2 (0.1)	2 (0.2)	0
Main occupation			
None	160 (6.7)	68 (7.0)	92 (6.4)
Employed or independent	323 (13.4)	161 (16.7)	162 (11.3)
Student	1810 (75.4)	701 (72.6)	1109 (77.2)
Homemaker	101 (4.2)	31 (3.2)	70 (4.9)
Other	8 (0.3)	4 (0.4)	4 (0.3)
Main occupation modality			
Face-to-face	903 (42.1)	341 (39.3)	562 (44.0)
Remote or virtual	698 (32.6)	320 (36.9)	378 (29.6)
Both	542 (25.3)	206 (23.8)	336 (26.3)
Missing data	259	NA	NA
Does not have own bedroom			
All	864 (36.0)	339 (35.1)	525 (36.5)
Missing data	4	NA	NA
Mother has received mental health treatment			
All	273 (11.4)	88 (9.1)	185 (12.9)
Missing data	373	NA	NA
Father has received mental health treatment			
All	100 (5.4)	27 (3.5)	73 (6.8)
Missing data	558	NA	NA

Abbreviation: NA, not available.

^a^ Other gender included missing and was not further subcategorized due to the small number.

^b^ Single includes single, separated, divorced, and widowed; not single includes partner, married, and cohabiting.

^c^ The categories complete and incomplete education were merged.

### Substance Use, Stressful Life Events, Sports and Arts Activities, Structural Social Capital, and Social Media Use

The lifetime use of substances, stressful life events during and before the past year, sports and arts activities in the past 30 days, structural social capital, and social media use in the past 30 days are summarized in Table 2. Overall, 1698 participants (70.7%) had used alcohol; 790 (32.9%), tobacco; 522 (21.7%), cannabis; and 304 (12.7%), sedatives. Participants had a mean (SD) of 2.98 (2.75) stressful life events in the past year and 7.02 (3.92) in their lifetime before that. In the past month, 778 (32.4%) had been involved in arts activities and 1132 (47.1%) in sports activities.

**Table 2.** Lifetime Consumption of Substances, Stressful Life Events, Structural Social Capital, and Sports, Arts, and Social Media Activities for the Total Sample and for Cases and Controls

Factor	Participants, No. (%)		
	Overall (n = 2402)	Cases (n = 1437)	Controls (n = 965)
Self-reported history of use			
Tobacco products			
Yes	790 (32.9)	525 (36.5)	265 (27.5)
Missing data	3	NA	NA
Alcoholic beverages			
Yes	1698 (70.7)	1066 (74.2)	632 (65.5)
Missing data	2	NA	NA
Cannabis			
Yes	522 (21.7)	349 (24.3)	173 (17.9)
Missing data	2	NA	NA
Cocaine			
Yes	136 (5.7)	84 (5.8)	52 (5.4)
Missing data	3	NA	NA
Amphetamine-type stimulants			
Yes	84 (3.5)	68 (4.7)	16 (1.7)
Missing data	2	NA	NA
Inhalants			
Yes	136 (5.7)	87 (6.1)	49 (5.1)
Missing data	3	NA	NA
Sedatives or sleep medication			
Yes	304 (12.7)	246 (17.1)	58 (6.0)
Missing data	3	NA	NA
Hallucinogens			
Yes	126 (5.2)	83 (5.8)	43 (4.5)
Missing data	2	NA	NA
Opioids			
Yes	21 (0.9)	15 (1.0)	6 (0.6)
Missing data	3	NA	NA
Other			
Yes	25 (1.0)	17 (1.2)	8 (0.8)
Missing data	12	NA	NA
Stressful life events, mean (SD), No.			
Past year	2.98 (2.75)	3.39 (2.93)	2.36 (2.33)
>1 y Ago	7.02 (3.92)	7.46 (3.86)	6.36 (3.91)
Missing data	53	NA	NA
Activities during the past 30 d			
Sports			
Yes	1132 (47.1)	626 (43.6)	506 (52.4)
Missing data	2	NA	NA
Arts			
Yes	778 (32.4)	503 (35.0)	275 (28.5)
Missing data	1	NA	NA
Structural social capital			
High	550 (22.9)	315 (21.9)	235 (24.4)
Medium	1367 (56.9)	841 (58.5)	526 (54.5)
Low	485 (20.2)	281 (19.6)	204 (21.1)
Missing data	54	NA	NA
High social media use			
Yes	1371 (57.2)	892 (62.2)	479 (49.7)
Missing data	10	NA	NA

Abbreviation: NA, not available.

### Univariable and Multivariable Models

Univariable and multivariable associations between depression and anxiety and the studied factors are presented in Table 3. In univariable analyses, different factors were associated with symptoms, with the highest ORs for lifetime use of sedatives and amphetamines. In the multivariable model, of all substance use categories, only use of sedatives (OR, 2.26 [95% CI, 1.65-3.14]) showed a significant association with depression and anxiety. Other factors associated with increased risk of depression and anxiety were female gender (OR, 1.99 [95% CI, 1.65-2.4]), more than 2 stressful life events in the previous year (OR, 1.67 [95% CI, 1.40-2.01]), more than 7 stressful life events before the previous year (OR, 1.52 [95% CI, 1.27-1.81]), arts activities in the past 30 days (OR, 1.22 [95% CI, 1.01-1.48]), and high social media engagement in the previous month (OR, 1.59 [95% CI, 1.34-1.89]). Sports activities in the past month was significantly associated with the absence of symptoms (OR, 0.80 [95% CI, 0.67-0.96]).

**Table 3.** Univariable and Multivariable Analyses of Factors Associated With Depression and Anxiety Symptoms

Factor	Univariable model		Multivariable model^a^	
	OR (95% CI)	*P* value	OR (95% CI)	*P* value
Age 20-24 y	1.01 (0.86-1.19)	.93	NA	NA
Female	2.13 (1.79-2.53)	<.001	1.99 (1.65-2.40	<.001
Sharing a bedroom	1.06 (0.90-1.26)	.48	NA	NA
Mother has received mental health treatment	1.47 (1.13-1.93)	.01	1.26 (0.95-1.68)	.12
≥2 Stressful life events in the past year	1.85 (1.56-2.19)	<.001	1.67 (1.40-2.01)	<.001
≥7 Stressful life events >1 y ago	1.72 (1.46-2.03)	<.001	1.52 (1.27-1.81)	<.001
Self-reported history of use				
Tobacco products	1.52 (1.27-1.82)	<.001	1.23 (0.98-1.56)	.08
Alcoholic beverages	1.51 (1.27-1.81)	<.001	1.15 (0.94-1.42)	.17
Cannabis	1.47 (1.20-1.80)	<.001	1.04 (0.80-1.35)	.76
Cocaine	1.09 (0.77-1.56)	.64	NA	NA
Amphetamine-type stimulants	2.95 (1.74-5.29)	<.001	1.53 (0.86-2.87)	.16
Inhalants	1.20 (0.84-1.74)	.31	NA	NA
Sedatives or sleep medication	3.23 (2.41-4.39)	<.001	2.26 (1.65-3.14)	<.001
Hallucinogens	1.31 (0.91-1.93)	.16	NA	NA
Opioids	1.69 (0.68-4.74)	.28	NA	NA
Sport activities in past 30 d	0.70 (0.59-0.82)	<.001	0.80 (0.67-0.96)	.02
Arts activities in past 30 d	1.35 (1.13-1.61)	<.001	1.22 (1.01-1.48)	.04
Structural social capital				
Medium	1.19 (0.98-1.46)	.15	NA	NA
Low	1.03 (0.80-1.32)		NA	
High social media use	1.67 (1.42-1.97)	<.001	1.59 (1.34-1.89)	<.001

Abbreviations: NA, not applicable; OR, odds ratio.

^a^ Results obtained from a multivariable logistic regression model. The sample size entering the multivariable model was 2370. The maximum condition index was 9.

### Interaction With Age Group

When all factors associated with the outcomes in the multivariable model at *P* < .05 were tested for their interaction with age group, we found an interaction effect only for the lifetime use of sedatives. The odds of having depression and/or anxiety symptoms associated with lifetime use of sedatives were higher among adolescents (OR, 6.54 [95% CI, 3.33-14.27]) than among young adults (OR, 2.54 [95% CI, 1.79-3.66]) (*P* = .01 for interaction) (eTable in Supplement 1).

## Discussion

In this case-control study, a number of different factors were associated with symptoms of anxiety and depression in young people in deprived urban neighborhoods in South America. In multivariable analysis, female gender, lifetime use of sedatives, and the experience of stressful life events before and during the past year were identified as independent factors associated with increased risk of anxiety and depression. The model also showed that the behavior of participants in the previous month was associated with symptoms. Stronger engagement with social media and arts activities were associated with symptoms of anxiety and depression and sports activities with the absence of these symptoms. The risk of symptoms associated with lifetime sedative use was higher in adolescents than in younger adults. Yet, all identified factors applied to both age groups and therefore across the age spectrum of youths as defined by the United Nations.^14^

### Comparison With the Literature

The identified factors associated risk of anxiety and depression are largely in line with the literature. Most of the literature, however, reflects research in high-income anglophone countries, and the studies were not specifically conducted in deprived urban areas where the risk for mental disorders is elevated. Our study assessed exclusively people living in such areas. Even within that context, the risk was higher in young female populations, as evidenced in other contexts,^24^ and in those with experience of more stressful life events. These result add to the general evidence that stressful life events, and particularly the experience of more violence, can impact mental health, as has been shown even in informal settlements in South America.^9,25^

In line with other studies, sedative use at young age was identified as a factor associated with increased risk of anxiety and depression. Sedative use has been associated with depression and poor academic achievement as well as problematic substance use later in life.^26^

Increasing evidence points to an association of intense social media engagement with worse youth mental health outcomes and an association of sports activities, particularly team sports, with better youth mental health outcomes.^27^ These processes appeared to be relevant also in deprived urban neighborhoods in South America. The multivariable analysis suggested that, regardless of other risk factors, strong engagement with social media may be harmful and participating in sports potentially beneficial for youth mental health.^27^

Evidence suggests that community arts activities can be beneficial for people’s life expectancy^28^ and their mental health.^29^ There are 2 potential reasons for the association of arts activities with more symptoms in this study. Young people with mental distress may be more motivated to seek and maintain arts activities.^30^ Also, a randomized clinical trial suggested that during the conditions of the pandemic, writing, which was included as an arts activity in the present study, had a negative influence on depressive symptoms.^31^ Writing is typically done alone, while other arts activities, such as performing theater, dance, or music, are commonly done in groups. A more detailed analysis of different arts activities is required to explore their exact role in influencing youth mental health.

### Implications

Associations in this observational study do not necessarily reflect unidirectional causal relationships. Participants with symptoms at the time of the assessment were more likely to have had symptoms already in the past, and those symptoms might have motivated them, for example, to use sedatives, to engage more with social media, or to participate in arts activities. At the same time, participants may have felt less encouraged or confident to participate in sports. The real influence may be in both directions; using sedatives may be both the result of mental distress and a factor driving it. Participation in sports activities is easier when there are no symptoms and may also help to prevent and overcome mental distress.

With this caveat, our findings on potential factors associated with anxiety and depression might inform policies in South America. Different factors appeared to be relevant independent of each other. Sociodemographic characteristics, such as female gender, are important. However, factors other than sociodemographic characteristics are more amenable to change. There are policies to prevent young people from substance use, although they are not universally implemented, and even when implemented, they often have a limited effect. Also, more than 80% of participants with symptoms in our study reported to have never used sedatives, so they would not be helped by policies for reducing sedative use.

This leaves 2 factors that may be addressed in policies: social media engagement and sports activities. A next step may be to develop and test strategies to reduce social media use in young people. With respect to sports activities, almost 50% of young people already participated in some sport in the preceding month. Increasing sports activities in deprived urban neighborhoods further may require funding for sports organizations and appropriate community spaces where young people can come together for sports activities.^32^

Finally, our findings suggest that studying youths following the United Nations’ definition of 15 to 24 years of age can be useful, as we found the same factors associated with risk of anxiety and depression in adolescents and young adults. Sedative use was associated with greater risk in adolescents but is relevant across the whole age spectrum.

### Strengths and Limitations

The study has several strengths. We recruited a large sample of participants in the challenging context of deprived urban areas, although recruitment was complicated because of the social restrictions during the COVID-19 pandemic. This was, to our knowledge, the first study exploring different factors associated with anxiety and depression in a population of young people across different countries in South America. We applied standardized assessment methods across the 3 cities. Considering generic symptoms of depression and anxiety made the results independent of specific diagnostic categories for mental disorders. Finally, while some factors, such as stressful life events, may be subject to memory and reporting bias, we focused on factors that may not be seen as automatically confounded with depression and anxiety.

The study also has several limitations. First, while we attempted to recruit widely within the defined inclusion criteria, the sample was not representative. The selection bias led to an overrepresentation of female participants and possibly also influenced the distribution of other characteristics and symptoms. It is possible that young people with mental distress were more motivated to participate in the study, but it is also conceivable that some young people were held back by symptoms of depression and anxiety or by shame of not functioning well. Overall, one can only speculate whether and, if so, how the selection bias affected factors other than gender and the prevalence of symptoms. However, the aim of the study was not to establish prevalence but to identify potential factors associated with anxiety and depression, and the analysis of associations of factors with outcomes was more robust against potential selection biases than estimates of prevalence. Second, all data reflect ratings of the participants, which may be inaccurate when reporting factual information. Third, different and changing social restrictions due to the COVID-19 pandemic during the initial phases of the data collection may have influenced the results. Finally, in a cross-sectional study, associations do not necessarily indicate causal relationships (limitations of multivariable analyses are given in eAppendix 2 in Supplement 1).

## Conclusions

In this case-control study of young people in deprived urban areas, female gender, stressful life events, substance use, arts activities, and social media engagement were associated with greater odds of depression and anxiety, while sport activities were associated with lesser odds. Initiatives to reduce substance use, reduce unhelpful social media engagement, and promote sports activities may be considered.

## Notes

Supplement 1. **eAppendix 1.** Recruitment strategies for participants in Bogotá, Buenos Aires and Lima **eMethods 1.** Adapted scale to measure stressful life events **eMethods 2.** Questions used to assess substance use **eAppendix 2.** Limitations of multivariable analyses **eTable.** Overall and Interactions

Supplement 2.
Data Sharing Statement
