# Supplementary material for: Mental Distress Among Youths in Low-Income Urban Areas in South America
Source: JAMA Netw Open. 2025 Mar 5;8(3):e250122. doi: 10.1001/jamanetworkopen.2025.0122 (PMC11883490; doi:10.1001/jamanetworkopen.2025.0122)
Supplement: Supplement 1. — eAppendix 1. Recruitment strategies for participants in Bogotá, Buenos Aires and Lima eMethods 1. Adapted scale to measure stressful life events eMethods 2. Questions used to assess substance use eAppendix 2. Limitations of multivariable analyses eTable. Overall and Interactions [file jamanetwopen-e250122-s001.pdf]

Supplementary Online Content

Gómez-Restrepo C, Diez-Canseco F, Brusco LI, et al. Mental distress among youths in deprived urban areas in South America. *JAMA Netw Open*. 2025;8(3):e250122. doi:10.1001/jamanetworkopen.2025.0122

eAppendix 1. Recruitment strategies for participants in Bogotá, Buenos Aires and Lima
eMethods 1. Adapted scale to measure stressful life events
eMethods 2. Questions used to assess substance use
eAppendix 2. Limitations of multivariable analyses
eTable. Overall and Interactions

This supplementary material has been provided by the authors to give readers additional information about their work.

© 2025 Gómez-Restrepo C et al. *JAMA Network Open*.

eAppendix 1. Recruitment strategies for participants in Bogotá, Buenos Aires and Lima

Bogotá

A partnership was established with the local Secretary of Education to recruit adolescents in 13 schools, and with the *Servicio Nacional de Aprendizaje* SENA (National Learning Service) to recruit young adults in 14 educational centres. Three different strategies were used as the study needed to adapt to the changing social restrictions due to the pandemic such as preventive isolation, remote education, and progressive return to normality in educational institutions. Initially, we conducted a virtual recruitment, where meetings were held with the directors of educational institutions to present the project and plan virtual meetings or obtain lists of students to identify potential participants. Subsequently, individual telephone calls were made with potential participants and their parents (for adolescents) for the screening, informed consent process, and completion of the questionnaires. In-person recruitment was conducted through face-to-face visits to educational institutions, during which the project was presented in all classrooms to identify potential participants, carry out the informed consent process and fill out the paper version of the questionnaires. Finally, we mixed virtual and in-person recruitment with in-person visits to educational institutions, during which the project was presented in all classrooms to identify potential participants. Subsequently, individual telephone calls were made with potential participants and their parents as above.

Buenos Aires

Young people were recruited from nine different organisations, also using three different strategies. In-person recruitment was facilitated through a collaboration with community organisations in the identified study areas. The organisations helped to identify and contact groups of eligible young people, carry out the screening, and select those who met the inclusion criteria. Additionally, there was an online recruitment strategy implemented through a link where potential participants could sign up and leave their contact information. This link was shared through the official communication channels of the University of Buenos Aires. The research team checked the inclusion criteria based on the available information, and contacted potential participants. When the informed consent process was conducted remotely, participants were informed about the study and completed the questionnaires virtually. Snowball recruitment was used. Young people who had already participated in the study were contacted and asked to invite people they knew who met the general inclusion criteria and might be interested in being part of the study.

Lima

Public and private institutions working with the target population were contacted and asked to invite potential participants. The institutions included 13 schools, five non-governmental organizations, two government programmes, two universities and one technical education centre. In addition, an important group of participants - mostly adolescents - were recruited through social media ads (Facebook and Instagram), targeting people living in the identified deprived areas. Potential participants provided their

© 2025 Gómez-Restrepo C et al. *JAMA Network Open*.

contact details through an online form. Subsequently, the research team contacted them to provide detailed information about the study and ascertain whether they met eligibility criteria. If they expressed interest, informed consent/assent was obtained from the participants, and consent from the responsible adults in the case of adolescents. Following the informed consent process, included participants completed the questionnaires virtually.

eMethods 1. Adapted scale to assess experience of stressful life events

Your Life experiences

Have any of the following events happened to you? Check the corresponding box depending on whether the event happened to you in the last year, more than a year ago or never. If an event happened to you more than once in different time periods, you can check more than one option.

		In the last year	More than a year ago	Never
1	Your parents/carers/brothers/sisters/children had a serious illness or injury (e.g. life-threatening or impacting their daily lives) or an operation			
2	Another person very close to you (e.g. a close friend)had a serious illness or injury (e.g. life-threatening or impacting your daily life) or an operation			
3	You had a serious illness or injury (e.g. life-threatening or impacting your daily life) or an operation			
4	You had a serious accident (e.g. an accident that required hospitalization)			
5	Your parents/siblings/partner/children had a serious accident (e.g. an accident that required hospitalization)			
6	Someone else very close to you had a serious accident (e.g. an accident that required hospitalization)			
7	You changed/moved house			
8	You were temporarily excluded from school			
9	You were permanently expelled from school			
10	You changed primary school			
11	You changed secondary school			
12	You were in care/foster home/children’s home			
13	Your family had continuing money problems, for example, they couldn't pay rent or bills			
14	Your mom/dad/brother/partner/child died			
15	Another person very close to you passed away			

© 2025 Gómez-Restrepo C et al. *JAMA Network Open*.

16	Your parents divorced or separated			
17	Your parents/carers/partner drank alcohol so often that it caused family problems			
18	You wereinvolved in a fire or natural disaster, for example, in a flood, earthquake, or hurricane			
19	You were the victim of a mugging or robbery			
20	Someone in your family was the victim of an mugging, robbery or burglary			
21	Someone very close to you was the victim of an mugging, robbery or burglary			
22	You were physically beaten and hurt (i.e. assaulted or attacked causing injury)			
23	You were the victim of another crime			
24	You moved here from another country or region because your parents/family were fleeing a difficult life (for example, due to war or violence)			
25	You've been homeless or lived on the street			
26	You cared for your mother, father, sibling, partner or child due to extended illness or disability			
27	You've been a victim of sexual harassment			
28	You have been the victim of physical or psychological bullying in person or virtual			
29	You've lost your job or had to drop out of school			
30	Are there any difficult experiences you've ever experienced?<br>Specify: ____			

© 2025 Gómez-Restrepo C et al. *JAMA Network Open*.

eMethods 2. Questions used to assess substance use

On the use or consumption of substances

We'll ask you some questions about your substance use experience throughout your life, as well as in the last three months. We will ask you about substances that can be smoked, ingested, inhaled, injected or consumed in pill form.

Some of the substances included may have been prescribed by a doctor. For this survey,we're not going to consider them if you took them as prescribed by your doctor.

Remember that all the information you provide to us is confidential and will not be shared with people outside the research team.

**1** Have you ever used the following substances in your life? (For non-medical use only)	**No**	**Yes**
1a Tobacco (cigarettes, chewing tobacco, cigars, etc.)		
1b Alcoholic beverages (beer, wine, pisco, rum, vodka, etc.)		
1c Marijuana (cannabis, joint, grass, hashish, etc.)		
1d Cocaine (paraic, pasta basica, stones, crack, etc.)		
1e Amphetamines and other stimulants (amphetamine, ecstasy, slimming pills, etc.)		
1f Inhalants (popper, glue/terokal, gasoline, paint solvents, etc.)		
1g Tranquilizers or sleeping pills (Valium/diazepam, Alprazolam/Xanax, Orfidal/Lorazepam, etc.)		
1h Hallucinogens (LSD, acid, mushrooms, tripe, ketamine, crystal, pepa etc.)		
1i Opioids (heroin, morphine, methadone, codeine etc.)		
1j Other type<br>Specify:____		

© 2025 Gómez-Restrepo C et al. *JAMA Network Open*.

eAppendix 2. Limitations of multivariable analyses

We used a multivariable analysis for identifying factors associated with symptoms. Multivariable analyses are widely established and commonly used in this type of exploratory studies. They have the advantages a) of testing whether the associations of factors that univariably are associated with symptoms may be better explained by other associations; b) of considering all relevant factors in one analysis; c) of thus avoiding problems of multiple testing; and d) of therefore reducing the risk of false positive results. However, they also have limitations. A major limitation is the risk of misleading negative findings. For example, taking amphetamines is univariably strongly associated with a higher probability of having symptoms. When in the multivariable analysis however other factors are also considered, there is no statistically significant correlation between taking amphetamines and symptoms anymore. The effect of taking amphetamines seems to disappear (because it may be better explained by the association of other factors with outcomes). Yet, one would surely be very cautious in interpreting this as evidence that taking amphetamines would not be harmful.
We still used this method as the most appropriate one, since we went for an exploratory analysis and not a hypothesis testing one. The exploratory nature of the design and analysis might also be regarded as a limitation of the whole study.

© 2025 Gómez-Restrepo C et al. *JAMA Network Open*.

eTable. Overall and Interactions

Variable	Descriptive table by variable			Stratified table by age group (Interaction between the variable and age group)						Estimated coefficient (β)				OR and interaction evaluation			
	**Overall**			**15-16**			**20-24**										
**Gender**	**Symptoms**	**No symptoms**	**Total**	**Symptoms**	**No symptoms**	**Total**	**Symptoms**	**No symptoms**	**Total**		**Estimated coefficient (β)**	**CI 95%**		**OR**	**CI 95%**		**p - value**
Female	1027	533	1560	471	232	703	556	301	857	**15 - 16**	0.95	0.68	1.22	2.58	1.97	3.38	0.053
Male	387	428	815	158	201	359	229	227	456	**20 - 24**	0.60	0.37	0.84	1.83	1.44	2.32	
Total	1414	961		629	433		785	528									
**Own bedroom**	**Symptoms**	**No symptoms**	**Total**	**Symptoms**	**No symptoms**	**Total**	**Symptoms**	**No symptoms**	**Total**		**Estimated coefficient (β)**	**CI 95%**		**OR**	**CI 95%**		**p - value**
No	525	339	864	284	192	476	241	147	388	**15 - 16**	0.00	-0.26	0.25	1.00	0.77	1.28	0.443
Yes	912	626	1538	361	243	604	551	383	934	**20 - 24**	0.13	-0.12	0.38	1.14	0.89	1.47	
Total	1437	965		645	435		792	530									
**Mother has received mental health treatment**	**Symptoms**	**No symptoms**	**Total**	**Symptoms**	**No symptoms**	**Total**	**Symptoms**	**No symptoms**	**Total**		**Estimated coefficient (β)**	**CI 95%**		**OR**	**CI 95%**		**p - value**
Yes	185	88	273	63	31	94	122	57	179	**15 - 16**	0.34	-0.12	0.83	1.41	0.88	2.29	0.810
No	1252	877	2129	582	404	986	670	473	1143	**20 - 24**	0.41	0.07	0.77	1.51	1.07	2.15	
Total	1437	965		645	435		792	530									

© 2025 Gómez-Restrepo C et al. *JAMA Network Open*.

**Life event score last year**	**Symptoms**	**No symptoms**	**Total**	**Symptoms**	**No symptoms**	**Total**	**Symptoms**	**No symptoms**	**Total**		**Estimated coefficient (β)**	**CI 95%**		**OR**	**CI 95%**		**p - value**
One or more events	1242	741	1983	575	337	912	667	404	1071	**15 - 16**	0.87	0.52	1.22	2.39	1.69	3.39	0.103
No event	195	224	419	70	98	168	125	126	251	**20 - 24**	0.51	0.22	0.80	1.66	1.25	2.22	
Total	1437	965		645	435		792	530									
**Life event score more than a year ago**	**Symptoms**	**No symptoms**	**Total**	**Symptoms**	**No symptoms**	**Total**	**Symptoms**	**No symptoms**	**Total**		**Estimated coefficient (β)**	**CI 95%**		**OR**	**CI 95%**		**p - value**
One or more events	1411	921	2332	634	414	1048	777	507	1284	**15 - 16**	1.07	0.28	1.91	2.92	1.33	6.78	0.666
No event	26	44	70	11	21	32	15	23	38	**20 - 24**	0.85	0.15	1.59	2.35	1.16	4.89	
Total	1437	965		645	435		792	530									
**Life event score last year (Median = 2)**	**Symptoms**	**No symptoms**	**Total**	**Symptoms**	**No symptoms**	**Total**	**Symptoms**	**No symptoms**	**Total**		**Estimated coefficient (β)**	**CI 95%**		**OR**	**CI 95%**		**p - value**
It is greater than or equal to median	1012	543	1555	470	248	718	542	295	837	**15 - 16**	0.71	0.44	0.97	2.03	1.55	2.64	0.364
It is less than to median	425	422	847	175	187	362	250	235	485	**20 - 24**	0.55	0.31	0.78	1.73	1.37	2.18	
Total	1437	965		645	435		792	530									
**Life event score more than a year ago (Median = 7)**	**Symptoms**	**No symptoms**	**Total**	**Symptoms**	**No symptoms**	**Total**	**Symptoms**	**No symptoms**	**Total**		**Estimated coefficient (β)**	**CI 95%**		**OR**	**CI 95%**		**p - value**

© 2025 Gómez-Restrepo C et al. *JAMA Network Open*.

It is greater than or equal to median	819	420	1239	323	168	491	496	252	748	**15 - 16**	0.47	0.21	0.72	1.59	1.24	2.06	0.384
It is less than to median	618	545	1163	322	267	589	296	278	574	**20 - 24**	0.61	0.38	0.84	1.85	1.47	2.33	
Total	1437	965		645	435		792	530									
**Tobacco products (cigarettes) a**	**Symptoms**	**No symptoms**	**Total**	**Symptoms**	**No symptoms**	**Total**	**Symptoms**	**No symptoms**	**Total**		**Estimated coefficient (β)**	**CI 95%**		**OR**	**CI 95%**		**p - value**
Yes	525	265	790	139	45	184	386	220	606	**15 - 16**	0.87	0.50	1.25	2.38	1.64	3.50	0.008
No	912	700	1612	506	390	896	406	310	716	**20 - 24**	0.29	0.06	0.52	1.34	1.07	1.68	
Total	1437	965		645	435		792	530									
**Alcoholic beverages (beer, wine, spirits, etc.) b**	**Symptoms**	**No symptoms**	**Total**	**Symptoms**	**No symptoms**	**Total**	**Symptoms**	**No symptoms**	**Total**		**Estimated coefficient (β)**	**CI 95%**		**OR**	**CI 95%**		**p - value**
Yes	1066	632	1698	396	209	605	670	423	1093	**15 - 16**	0.54	0.29	0.80	1.72	1.33	2.22	0.268
No	371	333	704	249	226	475	122	107	229	**20 - 24**	0.33	0.03	0.63	1.39	1.03	1.87	
Total	1437	965		645	435		792	530									
**Cannabis (marijuana, pot, grass, hash, etc.) c**	**Symptoms**	**No symptoms**	**Total**	**Symptoms**	**No symptoms**	**Total**	**Symptoms**	**No symptoms**	**Total**		**Estimated coefficient (β)**	**CI 95%**		**OR**	**CI 95%**		**p - value**
Yes	349	173	522	53	23	76	296	150	446	**15 - 16**	0.47	-0.05	1.02	1.60	0.95	2.79	0.836
No	1088	792	1880	592	412	1004	496	380	876	**20 - 24**	0.41	0.17	0.66	1.51	1.18	1.93	
Total	1437	965		645	435		792	530									

© 2025 Gómez-Restrepo C et al. *JAMA Network Open*.

**Cocaine (coke, crack, etc.) d**	**Symptoms**	**No symptoms**	**Total**	**Symptoms**	**No symptoms**	**Total**	**Symptoms**	**No symptoms**	**Total**		**Estimated coefficient (β)**	**CI 95%**		**OR**	**CI 95%**		**p -value**
Yes	84	52	136	10	7	17	74	45	119	**15 - 16**	-0.04	-1.11	1.10	0.96	0.33	3.00	0.789
No	1353	913	2266	635	428	1063	718	485	1203	**20 - 24**	0.11	-0.30	0.52	1.11	0.74	1.68	
Total	1437	965		645	435		792	530									
**Amphetamine type stimulants (speed, meth, ecstasy, ice etc.)**	**Symptoms**	**No symptoms**	**Total**	**Symptoms**	**No symptoms**	**Total**	**Symptoms**	**No symptoms**	**Total**		**Estimated coefficient (β)**	**CI 95%**		**OR**	**CI 95%**		**p -value**
Yes	68	16	84	13	1	14	55	15	70	**15 - 16**	2.19	0.29	5.94	8.93	1.33	380.18	0.242
No	1369	949	2318	632	434	1066	737	515	1252	**20 - 24**	0.94	0.34	1.60	2.56	1.41	4.94	
Total	1437	965		645	435		792	530									
**Inhalants (nitrous, glue, petrol, paint thinner, etc.)**	**Symptoms**	**No symptoms**	**Total**	**Symptoms**	**No symptoms**	**Total**	**Symptoms**	**No symptoms**	**Total**		**Estimated coefficient (β)**	**CI 95%**		**OR**	**CI 95%**		**p -value**
Yes	87	49	136	29	14	43	58	35	93	**15 - 16**	0.35	-0.34	1.08	1.42	0.71	2.93	0.553
No	1350	916	2266	616	421	1037	734	495	1229	**20 - 24**	0.11	-0.34	0.58	1.12	0.71	1.78	
Total	1437	965		645	435		792	530									

© 2025 Gómez-Restrepo C et al. *JAMA Network Open*.

**Sedatives/Sleeping Pills (Valium,Temazepam, Stilnox, etc.)**	**Symptoms**	**No symptoms**	**Total**	**Symptoms**	**No symptoms**	**Total**	**Symptoms**	**No symptoms**	**Total**		**Estimated coefficient (β)**	**CI 95%**		**OR**	**CI 95%**		**p - value**
Yes	246	58	304	86	10	96	160	48	208	**15 - 16**	1.88	1.20	2.66	6.54	3.33	14.27	0.013
No	1191	907	2098	559	425	984	632	482	1114	**20 - 24**	0.93	0.58	1.30	2.54	1.79	3.66	
Total	1437	965		645	435		792	530									
**Hallucinogens (LSD, acid, mushrooms, trips, Ketamine, etc.)**	**Symptoms**	**No symptoms**	**Total**	**Symptoms**	**No symptoms**	**Total**	**Symptoms**	**No symptoms**	**Total**		**Estimated coefficient (β)**	**CI 95%**		**OR**	**CI 95%**		**p - value**
Yes	83	43	126	7	3	10	76	40	116	**15 - 16**	0.46	-1.03	2.25	1.58	0.36	9.52	0.787
No	1354	922	2276	638	432	1070	716	490	1206	**20 - 24**	0.26	-0.15	0.69	1.30	0.86	1.99	
Total	1437	965		645	435		792	530									
**Opioids (heroin, morphine, codeine, etc.**	**Symptoms**	**No symptoms**	**Total**	**Symptoms**	**No symptoms**	**Total**	**Symptoms**	**No symptoms**	**Total**		**Estimated coefficient (β)**	**CI 95%**		**OR**	**CI 95%**		**p - value**
Yes	15	6	21	1	2	3	14	4	18	**15 - 16**	-1.09	-5.17	1.87	0.34	0.01	6.48	0.149
No	1422	959	2381	644	433	1077	778	526	1304	**20 - 24**	0.86	-0.30	2.29	2.37	0.74	9.92	
Total	1437	965		645	435		792	530									

© 2025 Gómez-Restrepo C et al. *JAMA Network Open*.

Sport activities last 30 days	Symptoms	No symptoms	Total	Symptoms	No symptoms	Total	Symptoms	No symptoms	Total		Estimated coefficient (β)	CI 95%		OR	CI 95%		p - value
Yes	626	506	1132	315	267	582	311	239	550	**15 - 16**	-0.51	-0.76	-0.26	0.60	0.47	0.77	0.111
No	811	459	1270	330	168	498	481	291	772	**20 - 24**	-0.24	-0.47	-0.01	0.79	0.63	0.99	
Total	1437	965		645	435		792	530									
**Artistic activities last 30 days**	**Symptoms**	**No symptoms**	**Total**	**Symptoms**	**No symptoms**	**Total**	**Symptoms**	**No symptoms**	**Total**		**Estimated coefficient (β)**	**CI 95%**		**OR**	**CI 95%**		**p - value**
Yes	503	275	778	283	143	426	220	132	352	**15 - 16**	0.47	0.21	0.73	1.60	1.23	2.07	0.079
No	934	690	1624	362	292	654	572	398	970	**20 - 24**	0.15	-0.11	0.41	1.16	0.90	1.50	
Total	1437	965		645	435		792	530									
**Structural social capital**	**Symptoms**	**No symptoms**	**Total**	**Symptoms**	**No symptoms**	**Total**	**Symptoms**	**No symptoms**	**Total**		**Estimated coefficient (β)**	**CI 95%**		**OR**	**CI 95%**		**p - value**
High	315	235	550	164	131	295	151	104	255	**15 - 16**	0.03	-0.30	0.35	1.03	0.74	1.42	0.552
Medium	841	526	1367	362	222	584	479	304	783	**20 - 24**	0.15	-0.12	0.43	1.16	0.88	1.53	
Low	281	204	485	119	82	201	162	122	284								
Total	1437	965		645	435		792	530									
**Social use media**	**Symptoms**	**No symptoms**	**Total**	**Symptoms**	**No symptoms**	**Total**	**Symptoms**	**No symptoms**	**Total**		**Estimated coefficient (β)**	**CI 95%**		**OR**	**CI 95%**		**p - value**
High*	892	479	1371	415	213	628	477	266	743	**15 - 16**	0.63	0.38	0.89	1.88	1.46	2.43	0.207

© 2025 Gómez-Restrepo C et al. *JAMA Network Open*.

Low	541	485	1026	229	221	450	312	264	576	**20 - 24**	0.42	0.1 9	0.6 5	1.52	1. 21	1.91	
Total	1433	964		644	434		789	530									
* Hight: It is greater than or equal to median (Median = 3)																	

© 2025 Gómez-Restrepo C et al. *JAMA Network Open*.
