# Supplementary material for: Mental Distress Among Youths in Low-Income Urban Areas in South America
Source: JAMA Netw Open. 2025 Mar 5;8(3):e250122. doi: 10.1001/jamanetworkopen.2025.0122 (PMC11883490; doi:10.1001/jamanetworkopen.2025.0122)
Supplement: Supplement 2. — Data Sharing Statement [file jamanetwopen-e250122-s002.pdf]

Data Sharing Statement

Gómez-Restrepo. Mental Distress Among Youths in Deprived Urban Areas in South America. *JAMA Netw Open*. Published March 05, 2025. doi:10.1001/jamanetworkopen.2025.0122

Data
Data available: Yes
Data types: Deidentified participant data
How to access data: The dataset analysed during the current study will be available beginning 9 months and ending 36 months following article publication to researchers on reasonable request from Professor Victoria Bird (v.j.bird@qmul.ac.uk).
When available: beginning date: 07-16-2025, end date: 07-16-2028

Supporting Documents
Document types: None

Additional Information
Who can access the data: Researchers who proposed use of the data has been approved.
Types of analyses: Specified purpose
Mechanisms of data availability: After approval of a proposal with a signed data access agreement.
